# Superhydrophobic Terrestrial Cyanobacteria and Land Plant Transition

**DOI:** 10.3389/fpls.2022.880439

**Published:** 2022-05-24

**Authors:** Wilhelm Barthlott, Burkhard Büdel, Matthias Mail, Klaus Michael Neumann, Dorothea Bartels, Eberhard Fischer

**Affiliations:** ^1^Nees Institute for Biodiversity of Plants, University of Bonn, Bonn, Germany; ^2^Department of Biology, University of Kaiserslautern, Kaiserslautern, Germany; ^3^Karlsruhe Nano Micro Facility and Institute of Nanotechnology, Karlsruhe Institute of Technology, Eggenstein-Leopoldshafen, Germany; ^4^Botanical Gardens, University of Bonn, Bonn, Germany; ^5^Institute of Molecular Biology and Biochemistry of Plants, University of Bonn, Bonn, Germany; ^6^Department of Biology, University of Koblenz-Landau, Koblenz, Germany

**Keywords:** evolution, *Hassallia*, key innovations, surface science, wettability, water repellency, chemical heterogeneity

## Abstract

Plants and other organisms have evolved structures and mechanisms for colonizing land since the Early Ordovician. In this context, their surfaces, the crucial physical interface with the environment, are mainly considered barriers against water loss. It is suggested that extreme water repellency (superhydrophobicity) was an additional key innovation for the transition of algae from water to land some 400 mya. Superhydrophobicity enhances gas exchange on land and excludes aquatic competitors in water films. In a different context, in material science and surface technology, superhydrophobicity has also become one of the most important bioinspired innovations enabling the avoidance of water films and contamination. Here, we present data for an extremely water-repellent cyanobacterial biofilm of the desiccation tolerant *Hassallia byssoidea* providing evidence for a much earlier prokaryotic Precambrian (ca. 1–2 bya) origin of superhydrophobicity and chemical heterogeneities associated with land transition. The multicellular cyanobacterium is functionally differentiated in a submerged basal hydrophilic absorbing portion like a “rhizoid” and an upright emersed superhydrophobic “phyllocauloid” filament for assimilation, nitrogen fixation, and splash dispersed diaspores. Additional data are provided for superhydrophobic surfaces in terrestrial green algae and in virtually all ancestral land plants (Bryophytes, ferns and allies, *Amborella*, *Nelumbo*), slime molds, and fungi. Rethinking of superhydrophobicity as an essential first step for life in terrestrial environments is suggested.

## Introduction

In many land-living organisms, extreme water repellence (superhydrophobicity: surface contact angle of water droplets > 150°) is one of the most prominent features. Superhydrophobicity was mentioned for the fern *Adiantum* (meaning “unwettable”) by Aristotle and Theophrastus, for Lotus (*Nelumbo*) in the Bhagavad-Gita, or for cabbage by Galileo Galilei (Barthlott et al., 2016a; Zeisler-Diehl et al., 2018). In physics and material science, publications about the self-cleaning principle of superhydrophobic plants (Barthlott et al., 2017) have led to a vast number of publications in the field of wetting and self-cleaning (“lotus effect”) of technical surfaces (Yan et al., 2011; Barthlott et al., 2016b; Bormashenko, 2017; Bhushan, 2018).

**FIGURE 1:**
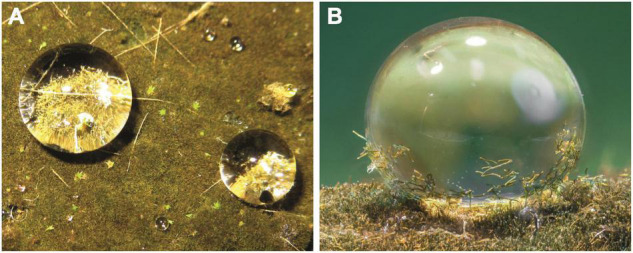
*Hassallia byssoidea* (Cyanobacteria). **(A)** Water droplets on the superhydrophobic biofilm. **(B)** Single droplet with a high contact angle and fragmented *Hassallia* filaments attached for splash dispersal.

**FIGURE 2:**
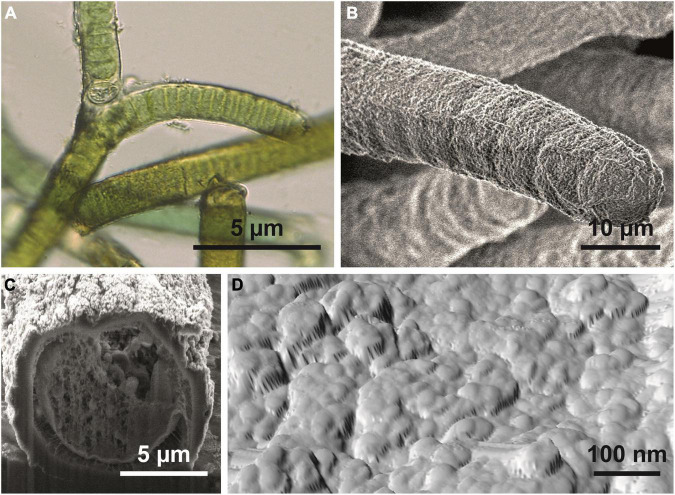
*Hassallia byssoidea* (Cyanobacteria). **(A)** Emersed superhydrophobic filaments showing false branching under a light microscope. **(B)** Single filament; the SEM image reveals the granular microstructure of the sheet. **(C)** Filament in cross section. **(D)** AFM image of the filament surface showing the system of semiglobular nanostructures (diameter 50–100 nm) arranged in a larger cluster of approximately 250–400 nm diameter.

**FIGURE 3:**
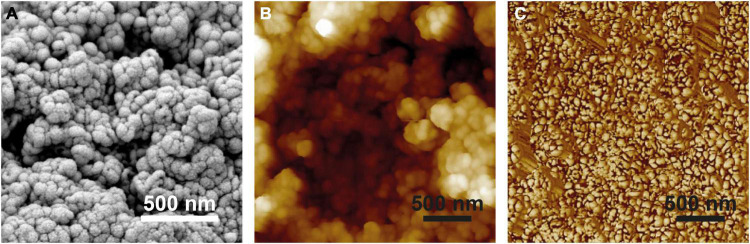
Surface structure of *Hassallia byssoidea* (Cyanobacteria). **(A)** SEM image of an air dried *H. byssoidea* fragment sputtered with a thin gold layer. **(B)** Topography (Height signal) of an *H. byssoidea* fragment measured by AFM. In comparison with the SEM image, the AFM image shows exactly the same structures and dimensions. The surface shows clusters of semi globular nanostructures. **(C)** Phase signal of the same AFM measurement as shown in **(B)**. Different colors represent different phase angles and indicate different attractive forces in these areas. This is a first hint for a chemical heterogeneity of the surface.

**FIGURE 4:**
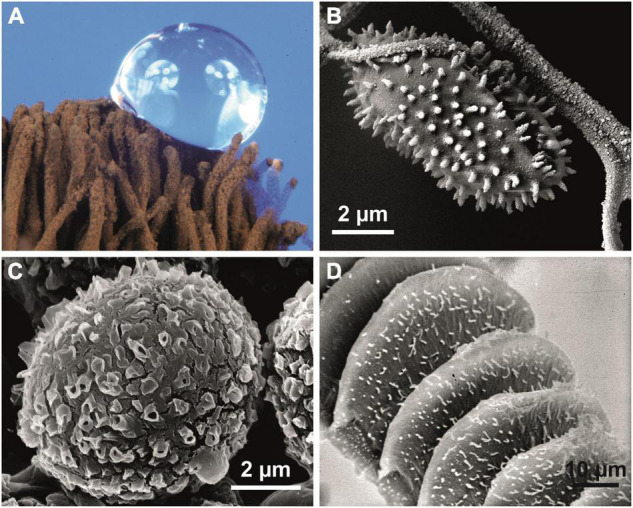
Superhydrophobic biological surfaces. **(A,B)** *Stemonitis* sp. (Mycetozoa); superhydrophobic reproductive structures of the slime mold. **(A)** Capillitium with water droplet. **(B)** Structured capillitium fibers and attached spinulose spore. **(C)** *Desmococcus olivaceus* (Chlorophyta); ornamented hydrophobic cells from a terrestrial biofilm. **(D)** The bonfire moss *Funaria hygrometrica* (Bryophyta) produces wax crystals only on its reproductive structures (peristome). Wax crystals are characteristic of the water repellency of all hydrophobic green plants.

**FIGURE 5:**
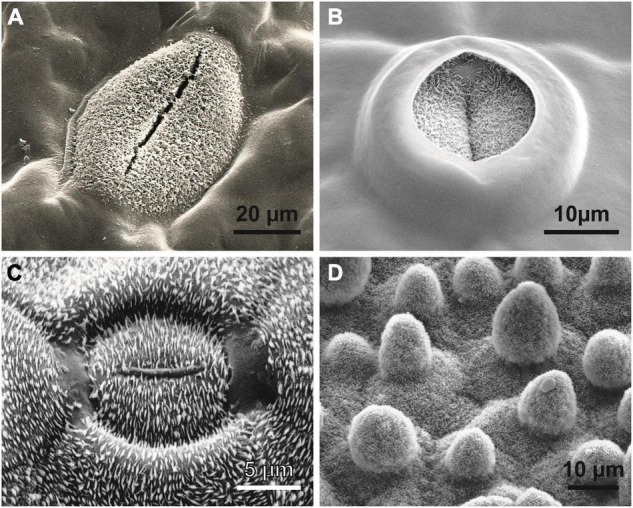
Wax crystals causing superhydrophobicity in vascular plants. **(A)** The hydrophilic tropical fern ally *Tmesipteris* (Psilotales). Similar to *Psilotum* and some *Selaginella* species, only the guard cells of the stomata are superhydrophobic to guarantee gas exchange under humid conditions. **(B)** The same phenomenon can be observed in the ancestral angiosperm *Amborella trichopoda* (Amborellaceae). In modern angiosperms, a high diversity of wax patterns and epidermal cell shapes can be observed, such as **(C)** the “electromagnetic field line” wax crystal pattern surrounding each stoma of the lily-of-the-valley *Convallaria majalis* (Monocots) or **(D)** the water- and dirt-repelling surface of the lotus *Nelumbo nucifera* (Eudicots), entirely covered by wax crystals; the sunken stoma in the center of the image is hardly recognizable.

Stable superhydrophobicity is restricted to biological surfaces and does not occur in natural abiotic materials (Barthlott et al., 2016a; Zeisler-Diehl et al., 2018). In flat technical surfaces (fluoropolymers such as PTFE), a contact angle of only ca. 120° is reached. In living organisms, hydrocarbon compounds in combination with a multiple hierarchical surface architecture (Figures 1–5), cause a much higher water repellency (contact angle of droplets up to 170°) (Zeisler-Diehl et al., 2018).

In vascular plants (Figure 5) and many insects, complex crystalline wax-like compounds (Barthlott et al., 1998) are usually responsible for surface hydrophobicity (Barthlott et al., 2017; Zeisler-Diehl et al., 2018). In some fungi (Ball et al., 2019), slime molds (Figures 4A,B), and Collembola (Yun et al., 2018), hydrophobic proteins (hydrophobins) seem to play the same role.

With a few exceptions, hydrophobicity is rather ignored in the comprehensive literature on cyanobacteria (Whitton, 2012; Schirrmeister et al., 2016), and the phenomenon is not discussed in the context of the eukaryotic algae to land plant transition (Bowles et al., 2020; Buschmann and Holzinger, 2020; Furst-Jansen et al., 2020). Our studies provide data for an extremely water-repellent cyanobacterial biofilm of the desiccation tolerant cyanobacterium *Hassallia byssoidea* which indicates a prokaryotic Precambrian (ca. 1–2 bya) origin of superhydrophobicity.

## Materials and Methods

### *Hassallia* Byssoidea Biofilm

A superhydrophobic “algal biofilm” has been developing since 2014 on a capillary mat covering a working bench in a tropical greenhouse of the Botanical Gardens of the University of Bonn (Germany). The mat was rinsed for watering of potted plants approximately once per day. The growth of a biofilm was observed over time; it was left undisturbed for 6 years and eventually covered an area of approximately 3 m^2^ in 2020. The biofilm (Figure 1A) was formed by the cosmopolitan heterocytic cyanobacterium *Hassallia byssoidea* (Tolypothrichaceae), associated with a few specimens of the moss *Philonotis hastata* (Bartramiaceae) (Komárek and Anagnostidis, 2007).

The *Hassallia* biofilm remained completely dry when rinsed with water. The contact angle of droplets on the biofilm (Figure 1B) was ca. 150°, measured using a contact angle measurement system (SCA 2.02, DataPhysics Instruments GmbH) and a small piece of the *Hassallia* covered capillary mat. To investigate the long term behavior of the hydrophobic surfaces under water, small portions (ca. 10 × 10 cm^2^) of the biofilm were excised and submersed in water (tap water, depth ca. 5 cm).

### Scanning Electron Microscopy

To explore the surface structures, responsible for the superhydrophobicity, different microscopy techniques were used. Scanning Electron Microscopy (SEM) (Auriga 60, Zeiss AG) images were made of air dried and critical point dried [dehydrated with ethanol and dried in a critical point dryer (CPD 020, Balzers-Pfeifer GmbH)] *Hassallia* samples. To check a possible influence on the surface structure by sputtering the samples with a thin gold layer (thickness 20 nm, Sputter Coater 108 auto, Cressington), samples of both types (air dried and critical point dried) were imaged with and without gold layer. Further, the influence of chloroform on the surface structure was investigated. Untreated samples were submerged in chloroform for about 30 min to monitor a possible influence on the surface. Those samples have also been analyzed using SEM with and without gold layer. Finally a comparison was made of superhydrophobic and wetted *Hassallia* samples.

Further, the cross section of *Hassallia* was investigated by cutting a critical point dried sample using a focused ion beam (FIB) system (Auriga 60, Zeiss AG) and imaging the freshly cut sample by SEM.

### Atomic Force Microscopy

Besides the SEM investigations, an untreated superhydrophobic *Hassalia* sample was analyzed using Atomic Force Microscopy (AFM). A small piece was excised from the *Hassallia* covered mat and placed in the AFM (Dimension ICON, Bruker) and scanned.

## Results

### Superhydrophobicity of the *Hassallia* Biofilm

Contact angle measurements confirmed the superhydrophobicity of the *Hassallia* biofilm. Color images of the droplets showed, that these water droplets serve as efficient dispersal agent due to the adhesion of *Hassallia* filaments (Figure 1B).

The investigation of the long term behavior of the submerged parts of the mats covered with *Hassallia* showed that the surface remained dry, retained a reflecting silvery layer of air for approximately 24 h and subsequently became completely wetted after 35–45 h under water. When the biofilm samples were removed from water and exposed again to air, the superhydrophobicity was reversed within 26–38 h.

The *Hassallia* biofilm is differentiated into an aerophytic upper “emersed” portion with superhydrophobic filaments and a hydrophilic basal “submerged” part of slightly paler filaments attached to the substratum.

### Microscopic Structures

The microscopic analysis revealed that the *Hassallia* filaments (Figures 2A,B) were covered by a 500–900 nm thick mucilaginous fibrous sheath (Figure 2C) that has a rough surface structure under dry conditions (Figure 2B). The AFM analysis (Figures 2D, 3) of untreated superhydrophobic *Hassallia* showed semiglobular nanostructures (diameter 50–100 nm) arranged in larger clusters of approximately 250–400 nm diameter (Figures 2D, 3), thus forming the hierarchical structure responsible for the extreme water repellence found in many eukaryotes. Additionally, the analysis of the AFM phase signal indicated nanoscopic chemical heterogenous spots in the semiglobular structures (Figure 3C). The different colors in the phase image represent different phase angles, often meaning different attractive forces, and hence different materials or chemical compositions in these areas. Such chemical heterogenous spots possibly stabilize the air-water interface, like in the floating fern *Salvinia* (Barthlott et al., 2010).

### Aerial Sheath Surface

The chemical composition and microstructure of the aerial sheath surface are not altered in either untreated or critical-point-dried specimens (see Supplementary Figure 1). This seems to be crucial for the formation of superhydrophobicity. A wax cover similar to that in green plants (Figures 5A–D) can be excluded, as no structural changes occurred after extensive treatment with chloroform or other solvents (Supplementary Figure 1). Hydrophobic proteins (“hydrophobeins”) known from other bacterial structures, including spores (Wiencek et al., 1990), may be responsible for the superhydrophobicity. Similar covers are known, e.g., from fungi (Ball et al., 2019), mycetozoa (Figures 4A,B), and springtails (Yun et al., 2018).

## Discussion

The superhydrophobicity of the aerial *Hassallia* surface is based on its hydrophobic chemical composition in hierarchical combination with the nanostructure of the cell surface plus the trichome-like morphology of the whole organism (Barthlott et al., 2017). Such a structural hydrophobicity (Wu et al., 2020) may play a role in the rain-repelling biocrusts like in Australia. Further, technical aluminum or copper oxide surfaces or superhydrophobic technical materials coated with synthesized nanoparticles look very similar (Yan et al., 2011).

In a survey of the superhydrophobic properties of terrestrial biocrusts in Central Europe, we additionally identified *Apatococcus lobatus* and *Desmococcus olivaceus* (both Trebouxiophyceae, Chlorophyta), common green algae in water-repellent biofilms on bark, rocks or buildings (Krienitz, 1995; Bertsch, 1966). In *Apatococcus* biofilms, a contact angle of ca. 155° was measured, and in *Desmococcus*, a slightly larger contact angle of ca. 160° was found. This might be attributed to the conspicuous spinulose surface architecture of *Desmococcus* (Figure 4C).

Superhydrophobicity is known in fungi and slime molds (Figures 4A,B), lichens (Shirtcliffe et al., 2006) and the earliest clades of arthropods (Wagner et al., 1996; Barthlott et al., 2016a) and other groups of animals. In embryophytic green plants, this property is caused by superhydrophobic wax crystals, most commonly non-cosan-10-ol-tubes (Figures 5A–C).

Land plants emerged in a Middle Cambrian-Early Ordovician interval from streptophytic green algae (Bowles et al., 2020; Buschmann and Holzinger, 2020; Furst-Jansen et al., 2020) approximately 400 mya, and we have demonstrated superhydrophobicity for the terrestrial streptophytic green algae *Apatococcus* and *Desmococcus*. However, the results obtained for *Hassallia* suggest that superhydrophobicity evolved with the conquest of land in autotrophic Precambrian prokaryotes, possibly ca. 1–2 bya, much earlier than expected before.

The change from a hydrophilic to a superhydrophobic state is rather common in aquatic green plants: submersed leaves or shoots, such as the inflorescence stalks of *Myriophyllum*, become hydrophobic within a few hours due to the formation of wax when growing out of water (Barthlott et al., 2017). However, the reverse change from a hydrophobic to a hydrophilic state has not been reported: submersed superhydrophobic plants maintain an air film, sometimes connected with a plastron-like function, as in *Triticum* (Konnerup et al., 2017) or *Salvinia* (Barthlott et al., 2010).

The basal “rhizoidal” filaments guarantee the water uptake and the superhydrophobic “phyllocauloids” are involved inassimilation, nitrogen fixation by heterocytes, and splash dispersal. A comparable phenomenon was indicated for Australian cyanobacterial biocrusts (Williams et al., 2014), where *Symplocastrum purpurascens* patches were differentiated into a wettable, substratum-attached lower section and an upper, somehow water-repellent section with upright bundles of filaments.

All these data suggest that superhydrophobicity may have been an essential prerequisite for the Precambrian escape of cyanobacteria from water with its many prokaryotic associates for colonizing the new dry habitat *“land”* without competitors. Superhydrophobicity is essential to avoid water films, thus allowing improved gas exchange. Even recent tree ferns in wet cloud forests such as *Cibotium glaucum* (Barthlott et al., 2016a,^2017^) or semiaquatic lotus plants (*Nelumbo*, Figure 4D) use this mechanism. In *Hassallia*, superhydrophobicity is the basis for an early rain-operated splash dispersal mechanism on land (Figure 1B), and, like in antifungal substances, superhydrophobicity may be an outer-perimeter defense mechanism (lotus effect) against contamination and pathogenic spores (Zeisler-Diehl et al., 2018). Based on these assumptions and our SEM research on the surface structures of ca. 20 000 species [surveys in Barthlott et al. (2017)], we systematically analyzed bryophytes: at least gas-exchange structures and reproductive structures were observed in many species of Marchantiophyta, Anthoceratophyta, and Bryophyta (Figure 4D), as were superhydrophobic structures, such as in ancestral tracheophytes such as *Selaginella*, *Psilotum*, *Tmesipteris* (Figure 5A) and the most ancestral angiosperm *Amborella* (Figure 5B). Even in rainforest species, we found that at least the stomatal cells are usually superhydrophobic due to wax crystals. Ferns and fern allies show the full range of structural and chemical compounds. Some tree ferns (*Cibotium glaucum*) in moist-saturated cloud forest habitats have superhydrophobic frond surfaces entirely covered with wax (Barthlott et al., 2017). In many angiosperms, the gas exchange surfaces show elaborate stomatal wax formations, such as the magnetic field pattern (Barthlott et al., 2017) in the lily-of-the-valley *Convallaria* (Figure 5C). The unsurpassed example is the superhydrophobic complexly hierarchically structured self-cleaning (“lotus effect”) leaves of semiaquatic *Nelumbo* (Figure 5D) with a contact angle up to > 170°, which led to the enormous interest in surface physics and innovations in material science of the last two decades.

All interactions between living organisms and their environment are controlled by the surface boundary layers as the crucial interface. Consequently, terrestrial organisms such as *Hassallia* had to evolve mechanisms to cope with the new environmental conditions outside of water. The genome of *Hassallia* (Das et al., 2015; Singh et al., 2015) has the capacity to encode several putative hydrophilin proteins and many response regulators typically involved in bacterial stress responses, but no genomic fragments that encode late embryogenesis abundant (LEA) proteins or dehydrin proteins, which are both closely correlated with dehydration tolerance in angiosperm plants, ferns and mosses, have been identified (Oliver et al., 2020).

The evolution of hydrophobicity in *Hassallia* (Tolypotrichaceae) does not necessarily correspond with the evolutionary timeline of Cyanobacteria in general. According to Warshan et al. (2018) the family Tolypotrichaceae probably evolved after the “endoxymnbiotic event” about 1 bya. Phylogentic trees of this clade are published (Hauer et al., 2014). Additionally, there is evidence, that terrestrial cyanobacterial biofilms probably existed since 2 bya (Beraldi-Campesi and Retallack, 2016)—and we provided evidence that superhydrophobicity is a prerequisite for their existence.

*Hassallia* evolved with its chemical heterogeneity already a differentiation in substrate-bound hydrophilic “rhizoid”-like filaments for water and nutrient uptake and superhydrophobic “phyllocauloid”-like filaments for assimilation, nitrogen fixation, and production of splash dispersed diaspores. Thus, this multicellular desiccation tolerant cyanobacterium (Herrero et al., 2016) already exhibits basic principles of eucaryotic green land plants like Bryophytes. In this context it is remarkable, that in the dry state many desiccation tolerant mosses are superhydrophobic to avoid a premature re-vitalization by ephemerous water contact: we found even dry leaves of the angiosperm desiccation tolerant resurrection plant *Craterostigma plantagineum* (Linderniaceae) (Giarola and Bartels, 2015) to be water repellent.

Our data suggest that we need to rethink the role of superhydrophobicity and consider it as a possible key evolutionary step enabling photoautotrophic organisms to avoid water films for the ease of gas exchange and easing the transition of life from water to land for aquatic heterotrophic competitors approximately between 1 and 2 billion years ago.

## Data Availability Statement

The original contributions presented in the study are included in the article/Supplementary Material, further inquiries can be directed to the corresponding author.

## Author Contributions

WB: concept of the research, analysis, and interpretation of biological superhydrophobic surfaces. BB: ecology, phylogeny, ecophysiology and taxonomy of cyanobacteria, and biofilms. MM: scanning electron microscopy and focused ion beam, atomic force microscopy, photography, and background in surface physics. KN: experimental, wettability and cultivation of living cyanobacterial material. DB: analyze the published *Hassallia* genome sequence to identify dehydration-related genes. EF: taxonomy, phylogeny, and ecology of cyanobacteria and vascular plants. All authors contributed to the article and approved the submitted version.

## Conflict of Interest

The authors declare that the research was conducted in the absence of any commercial or financial relationships that could be construed as a potential conflict of interest.

## Publisher’s Note

All claims expressed in this article are solely those of the authors and do not necessarily represent those of their affiliated organizations, or those of the publisher, the editors and the reviewers. Any product that may be evaluated in this article, or claim that may be made by its manufacturer, is not guaranteed or endorsed by the publisher.
