# Supplementary material for: Superhydrophobic Terrestrial Cyanobacteria and Land Plant Transition
Source: Front Plant Sci. 2022 May 24;13:880439. doi: 10.3389/fpls.2022.880439 (PMC9173694; doi:10.3389/fpls.2022.880439)
Supplement: Supplementary file 1 [file Data_Sheet_1.pdf]

Supplementary Material

Superhydrophobic terrestrial cyanobacteria and land plant transition

Analysis of the hydrophobic surface of *H. byssoidea*

To confirm the nature of hydrophobicity, in particular whether the nanostructure, like in most green plant surfaces, consists of waxes, and how the structure behaves under different conditions, several samples were treated differently and afterwards analyzed by SEM (Auriga 60, Zeiss AG). The structures are close the SEM resolution. The results are shown in Fig. S1. For each treatment, two images are shown. In one case, the sample was brought into the SEM directly after the treatment to exclude further influences to the surface e.g. by the sputter coating process. In the other case, a thin gold layer (thickness 20nm, Sputter Coater 108auto, Cressington) was deposited to the surface in order to enhance the conductivity. To prepare the samples, they were either air dried or dried using CPD (dehydrated with ethanol and dried in a critical point dryer (CPD 020, Balzers- Pfeifer GmbH)) as descried in the method section. The comparison of these two preparation methods allows, on the one hand to analyze the influence of the solvent used for the CPD, on the other hand deformations due to the air drying are excluded. As no change between CPD dried and air dried samples was found, other samples were treated with chloroform (samples were submerged for about 30 minutes). As can be seen in Fig. S1, no significant change due to the chloroform treatment could be seen. This led to the assumption that the surface structure consists of material other than waxes and needs to be further analyzed.

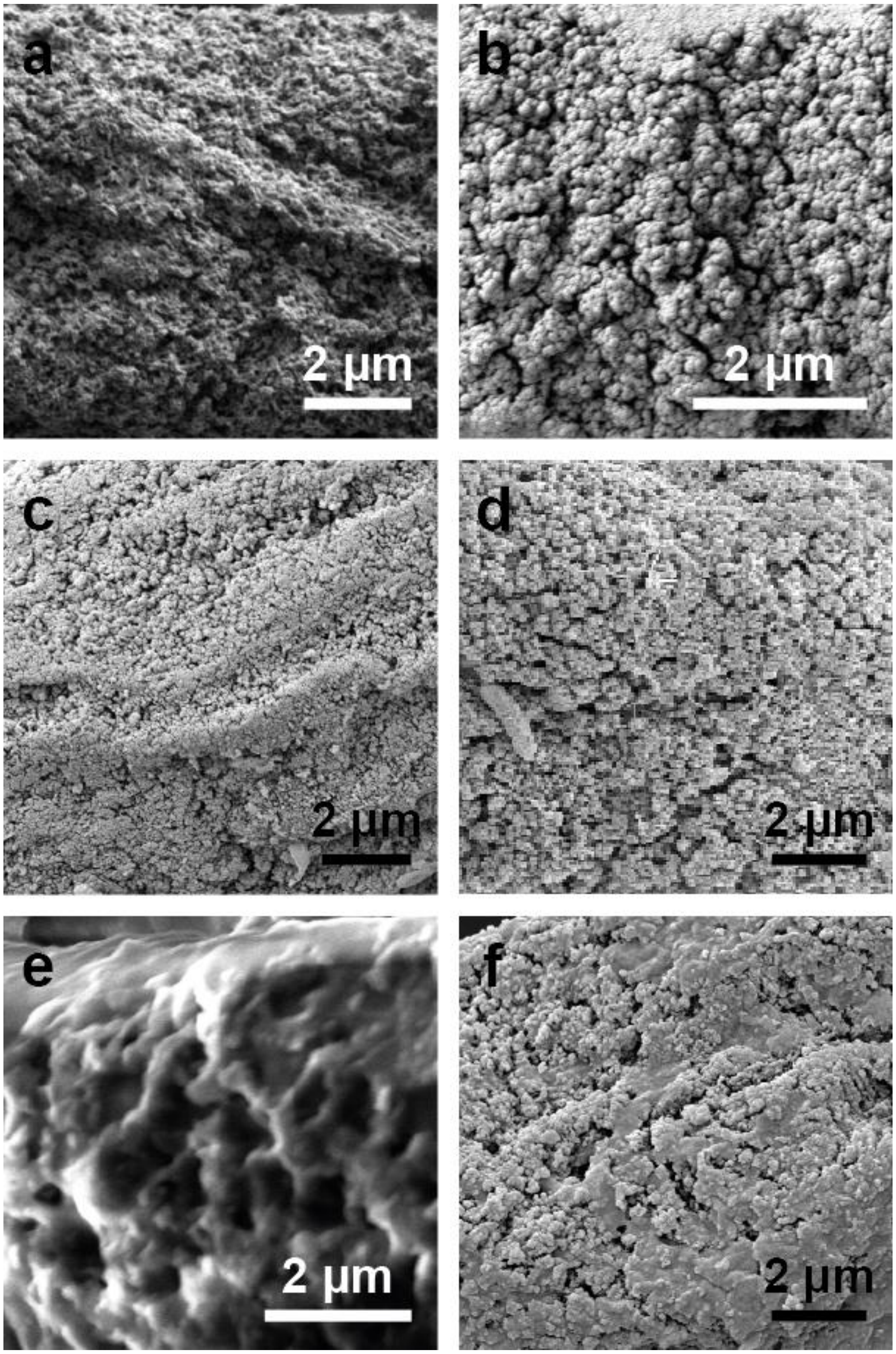

Fig. S1: Surfaces of *H. byssoidea* after different treatments. Each row represents one treatment. The left images show samples without an additional gold layer, in the right images a thin gold layer was deposited on the surface to enhance the conductivity. **a)** and **b)** show images of the surface of air dried *H. byssoidea* fragments. **c)** and **d)** In this case the samples were critical point dried before they were analyzed by SEM. **e)** and **f)** shows the surface of *H. byssoidea* after treating it with chloroform.

Comparison of hydrophobic and hydrophilic *H. byssoidea* surface

As mentioned in the main text, *H. byssoidea* changes from superhydrophobic to hydrophilic and then back again. In order to investigate, whether a change of the surface structure might be responsible for the changing wettability, samples of both states were analyzed by SEM (Auriga 60, Zeiss AG). The preparation procedure is described in the Methods section. In Fig. S2 two sample images of these investigations are shown. No significant change in the surface structure could be seen between the superhydrophobic and the hydrophilic sample, so probably other mechanisms must be responsible for the changing wettability.

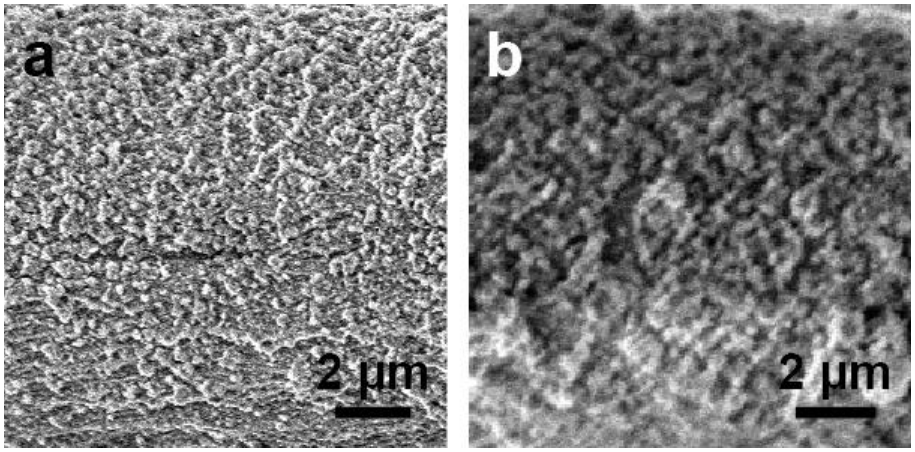

Fig. S2: Surface comparison of unwettable and wetted *H. byssoidea* fragments. Samples were shock frozen using liquid nitrogen and investigated without any coating. **a)** Surface of a superhydrophobic *H. byssoidea* fragment. **b)** Surface of a wetted (hydrophilic) *H. byssoidea* fragment. Between the two surfaces no significant difference was found.
